# Cardiac Amyloidosis: Expanding Awareness to Optimize Diagnosis

**DOI:** 10.1016/j.jacadv.2024.101087

**Published:** 2024-07-11

**Authors:** Michelle M. Kittleson

**Affiliations:** Department of Cardiologist, Smidt Heart Institute, Cedars-Sinai Medical Center, Los Angeles, California, USA

**Keywords:** cardiac amyloidosis, heart failure

**Figure:**
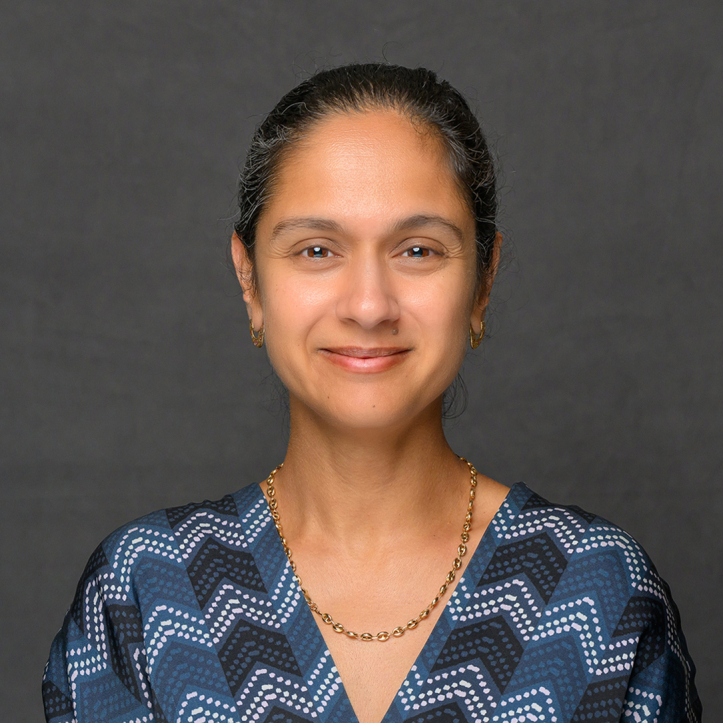

Cardiac amyloidosis results in a restrictive cardiomyopathy caused by extracellular deposition of proteins in the myocardium. The proteins have an unstable structure that causes them to misfold, aggregate, and deposit as amyloid fibrils. The origin of the misfolded protein in cardiac amyloidosis is typically either monoclonal immunoglobulin light chains (AL) from an abnormal clonal proliferation of plasma cells, or transthyretin amyloidosis (ATTR), a liver-synthesized protein that is normally involved in the transportation of the hormone thyroxine and retinol-binding protein.

ATTR amyloidosis is more common than AL amyloidosis and can be inherited as an autosomal dominant trait caused by pathogenic variants in the transthyretin gene *TTR* (ATTRv) or by the deposition of wild-type transthyretin protein (ATTRwt). Historically, median survival after diagnosis in untreated patients is poor: 2.5 years for ATTRv caused by the *TTR* Val122Ile (or pV142I) mutation, and 3.6 years for ATTRwt.^1^, ^2^, ^3^

Several critical advances in the diagnostic approach, coupled with approval of effective therapies, have elevated cardiac amyloidosis to a position of diagnostic prominence.^4^ First, imaging techniques and monoclonal protein testing now allow for accurate noninvasive diagnosis of ATTR cardiac amyloidosis^5^ without the need for confirmatory endomyocardial biopsies. Second, observational studies indicate that ATTR cardiac amyloidosis may be under-recognized in a significant proportion of patients with heart failure.^6^^,^^7^ Third, prompt implementation of therapeutic interventions, namely tafamidis, can improve survival, physical function, and/or quality of life.^8^^,^^9^

With these advances over the past decade, there has been a substantial increase in diagnosis of ATTR cardiac amyloidosis, with more patients diagnosed at an earlier stage of the disease with substantially lower mortality over time.^10^ The impact of increased awareness on prompt recognition, appropriate diagnosis, and timely treatment also can be inferred from comparison of outcomes in the 2 landmark clinical trials in ATTR cardiac amyloidosis. The Transthyretin Amyloidosis Cardiomyopathy Clinical Trial (ATTR-ACT) enrolled patients from December 2013 through August 2015.^9^ After 30 months, 42.9% of patients receiving placebo had died, compared with 29.5% of patients receiving tafamidis. In contrast, the Efficacy and Safety of AG10 in Subjects with Transthyretin Amyloid Cardiomyopathy (ATTRibute-CM) trial enrolled patients from April 2019 through October 2020 and, after 30 months, 24.2% of patients in the placebo group had died, compared with 17.8% of patients receiving acoramidis.^8^ The fact that patients in the placebo group in ATTRibute-CM had 30-month mortality that was lower than that of patients in the treatment group in the ATTR-ACT trial suggests improvements in other aspects of amyloid care over the ensuing almost 5 years between trial enrollments, including earlier diagnosis.

Currently, the only evidence-based, guideline-recommended, and FDA-approved therapy for ATTR cardiac amyloidosis is tafamidis,^9^ a TTR stabilizer, with promising clinical trial findings for acoramidis another stabilizer.^8^ Because TTR stabilizers inhibit the dissociation of the stable TTR tetramers into monomers, an essential step in fibril formation and subsequent tissue deposition, these agents do not reverse disease; once initiated, they only slow progression. Thus, early recognition and diagnosis of ATTR amyloidosis afford patients the greatest chance that treatment will favorably impact survival and prevent potentially irreversible loss of physical function and quality of life. In fact, only less symptomatic patients, those with New York Heart Association class 1 to 2 symptoms at initiation, had better outcomes when treated with tafamidis in the ATT-ACT trial.^9^

If the key to better outcomes is early diagnosis, and early diagnosis requires increased awareness, then a crucial factor to prompt diagnosis of amyloidosis is recognition of the typical affected demographic. Observational studies unequivocally demonstrate that ATTR amyloidosis is most commonly diagnosed in those over age over 70 years and more often in men than in women.^3^ However, this means that patients under 70 years of age, as well as women, may experience delays in diagnosis. Given this, a better understanding of the age and sex differences in clinical manifestations may reduce demographic diagnostic bias and allow for increased awareness and earlier treatment in typically underrepresented groups. In the context, the study by Mora-Ayestaran et al^11^ in this issue of *JACC: Advances* used THAOS (Transthyretin Amyloidosis Outcomes Survey) examines the age- and sex-specific clinical manifestations of ATTR cardiac amyloidosis in the hopes that this information could increase clinicians’ awareness and recognition of cardiac amyloidosis.

THAOS is the largest global, longitudinal, observational registry of patients with ATTR amyloidosis, initiated in 2007 and comprising 53 centers from 15 countries. The authors examined 1,251 patients with ATTRwt amyloidosis and made several important observations. First, while 17.1% of patients with ATTRwt amyloidosis were under 70 years of age, 23.3% of them had New York Heart Association functional class 3 to 4 symptoms. This suggests that younger patients were not diagnosed until they developed more advanced disease, perhaps due to less awareness of amyloidosis as a potential diagnosis in younger patients.

A second important observation is that while most patients with ATTRwt amyloidosis were men, the proportion of women increased with age from 4.1% in patients under 70 years of age to 14.3% in patients 90 years of age or greater. In fact, the longest median time from symptom onset to diagnosis was in women 70 years of age, with a delay in diagnosis of 5.2 years.

A third important finding was that women with ATTRwt amyloidosis had lower left ventricular wall thickness than men, though not when wall thickness was indexed by height. This observation suggests that a sex-specific wall thickness threshold for diagnosis may be necessary to avoid underdiagnosis of cardiac amyloidosis in women.

While these findings are compelling, the limitations of the current analysis must be addressed. Of the 1,251 patients included in the analysis, only 7 were women under 70 years of age and only 5 were women aged 90 years or older. So while numerical trends may be observed, further studies are needed to confirm these findings, especially as other studies have not demonstrated sex-specific differences in disease presentation, progression, or prognosis.^12^

Nonetheless, there are important lessons for clinicians from this analysis. If woman under the age of 70 presents with heart failure symptoms and increased left ventricular wall thickness on echocardiogram, particularly increased wall thickness indexed to height, clinicians should not discount a diagnosis of cardiac amyloidosis. Often, there are other helpful clinical clues suggesting the diagnosis, both cardiac (aortic stenosis, atrial fibrillation) and extra cardiac (sensory peripheral neuropathy, orthostatic hypotension, carpal tunnel syndrome, lumbar spinal stenosis).^13^

As with most areas of medicine, judgment and experience are key to providing the best care to patients with amyloidosis. Keeping an open mind to all diagnostic possibilities is essential; a patient’s presentation should be interpreted through the lens of their other medical history and in the case of amyloidosis, seemingly disparate neurologic or orthopedic manifestations may be part of a unifying diagnosis. As this study by Mora-Ayestaran et al demonstrates, these factors should not be discounted, even if the patient does not fit the typical demographic of age and sex.

## Funding support and author disclosures

The author has reported that they have no relationships relevant to the contents of this paper to disclose.
